# Influencing factors of weak grip strength and fall: a study based on the China Health and Retirement Longitudinal Study (CHARLS)

**DOI:** 10.1186/s12889-022-14753-x

**Published:** 2022-12-13

**Authors:** Hao Liu, Yunfei Hou, Hu Li, Jianhao Lin

**Affiliations:** Arthritis Clinic & Research Center, Peking University People’s Hospital, Peking University, 100044 Beijing, China

**Keywords:** Fall, Weak grip strength, China Health and Retirement Longitudinal Study (CHARLS), Risk factors

## Abstract

**Background:**

Fall is a major cause of mortality and cause a significant burden on the healthcare system and economic system. Weak grip strength signifies impaired function. Older people with weak grip strength are at a higher risk of death. China has the largest ageing population in the world today. This study aims to analyze the factors contributing to weak grip strength and fall among Chinese.

**Methods:**

This study analyzed data from the 2011 baseline and 2015 follow-up survey of the China Health and Retirement Longitudinal Study (CHARLS). To identify the risk factors of fall and weak grip strength, we used a stepwise multivariable logistic regression model and a least absolute shrinkage and selection operator (LASSO) regression model.

**Results:**

In the LASSO regression model, all the risk factors were not shrunken. In the stepwise logistic regression model, adjusted for gender, age, grip strength, depression, and chronic disease, we found that female (aOR = 1.376, 95% CI = 1.243–1.523; *P* < 0.001), history of ischemic stroke (aOR = 1.786, 95% CI = 1.263–2.524; *P* = 0.001), depression (aOR = 1.559, 95% CI = 1.396–1.742; *P* < 0.001), weak grip strength (aOR = 1.285, 95% CI = 1.105–1.494; *P* = 0.001), older age (aOR = 1.227, 95% CI = 1.163–1.294; *P* < 0.001), rheumatoid arthritis (aOR = 1.410, 95% CI = 1.270–1.560; *P* < 0.001), history of kidney disease (aOR = 1.383, 95% CI = 1.136–1.682; *P* = 0.001) were factors associated with fall significantly. After further adjusting, we found the risk factors of weak grip strength included symptomatic knee osteoarthritis (aOR = 1.755, 95% CI 1.158–2.661; *P* = 0.008), living in rural area (aOR = 2.056, 95% CI 1.290–3.277; *P* = 0.002), depression (aOR = 1.523, 95% CI 1.116–2.078; *P* = 0.008), older age (aOR = 2.116, 95% CI 1.801–2.486; *P* < 0.001).

**Conclusion:**

From the study, we found that older age and depression were risk factors of weak grip strength and fall. Weak grip strength was a risk factor of fall. Female, ischemic stroke, kidney disease, rheumatoid arthritis were risk factors of fall; living in rural area and symptomatic knee osteoarthritis were risk factors of weak grip strength.

**Supplementary Information:**

The online version contains supplementary material available at 10.1186/s12889-022-14753-x.

## Background

Fall is a major cause of bone fractures [1], restricted activity [2], and mortality [3]. Fall-related injuries cause a significant burden on the healthcare system and economic system [4]. There are many factors related with fall, including limitations of functional performance, pain, stiffness, impaired proprioception, and obesity [5–7]. Gender is also associated with fall. Women have higher incidence of fall than men (nearly twice as high) [7].

Physiological [8], psychological [9, 10], cognitive [11], and performance-based [12] assessments have been proposed to evaluate the potential fall risk. The risk factors of fall included age, disability, poor performance on physical tests, depressive symptom [9, 10]. Despite enormous fall-prevention researches, the prevalence of falls remains unchanged. Approximately one in three people over 65 years reported at least one fall each year [13]. This may also be due to risk factors that have never been well understood.

Weak grip strength signifies impaired function, further increasing the risk of injuries. There is evidence showing that those in the lowest quarter of grip strength were at over 1.5-fold the risk of death during follow-up compared to those in the highest quarter [14]. Vitamin B12 deficiency [15], older age [16–18], female [16–18],depression [17] ,physical activity [17], and work status [19] were potential risk factors of weak grip strength. The prevalence of weak grip strength was 34.4% in China [16]. However, few researchers studied the potential risk factors of weak grip strength involving in a large number of Chinese participants.

China has the largest ageing population and one of the highest ageing rates in the world today [20]. It is projected that the proportion of those aged 65 or older will increase from 7% of the population in 2010 to 26% in 2050 [21]. The old-age support ratio (defined as the number of prime-age adults aged 15 to 64 divided by the number of adults aged 65 or above) will drop from about 9.9:1 in 2010 to 2.3:1 in 2050 [21]. The population in China is ageing and ageing-related burden on society is serious. According to the above literature, falls and weak grip strength are two important ageing-related problems which can impair function and put enormous burden on economy or healthcare. The muscle mass and muscle strength of Asian are different from White [22]. Besides, bone microstructure of Chinese is also different from White [23, 24]. The rates of fall and hip fracture of Chinese women are lower than in white women [24]. The risk factors, which are closely associated with weak muscle strength and fall, are also probably different. Some studies assessed the potential factors [9, 10] but few researchers assessed the similar characteristics between fall and weak grip strength among Chinese in a study.

This study used data from the China Health and Retirement Longitudinal Study (CHARLS) to assess the factors associated with fall and weak grip strength and found similar characteristics between them to instruct preventions.

## Materials and methods

### Data

CHARLS is a longitudinal survey of the residents in mainland China aged 45 and older. The CHARLS baseline survey covered 150 countries/districts, 450 villages/urban communities across the country, involving 17,708 individuals in 10,257 households [20].

CHARLS was designed to provide detailed information about the health of the older Chinese populations (more than 45 years old). We designed a study to find the risk factors of fall and weak grip strength. The data of 2018 follow-up is incomplete, which is short of physical examination and some other healthy data in 2018 follow-up. Thus, this study used data from the 2011 baseline and 2015 follow-up survey of the CHARLS. We used 2011 data as baseline and merged 2013 data with 2015 data as a cohort survey. For grip strength related analysis, exclusion criteria were (1) no grip strength information at baseline; (2) weak grip strength at baseline survey; (3) no grip strength information at 2015 follow-up. For fall-related analysis, exclusion criteria were (1) no fall-related information at baseline survey; (2) falls at baseline survey; (3) no fall-related information at 2013 and 2015 survey. A total of 8437 participants were included in the part of “weak grip strength study”. There were a few dozen missing data in some variables, such as variables of chronic disease par. A total of 9284 participants were included in the part of “fall study”.

Ethical approval for all the CHARLS waves was granted from the Institutional Review Board at Peking University. The IRB approval number for the main household survey, including anthropometrics, is IRB00001052-11015.

### Outcomes

#### Grip strength

Trained examiners in CHARLS instructed people to hold the dynamometer and squeeze the handle for a few seconds. We both measure right and left-hand grip strength twice in each hand. If grip strength of both right and left hand are all less than the criteria (man < 30 kg, woman < 20 kg), it is defined as weak grip strength [15, 25].

#### Fall

Fall was participants self-reported outcome, which was assessed based on “Have you fallen down in the last two years?” If a participant gave the answer “yes”, he/she was defined as those who have been fallen.

### Description of variables [26, 27]

#### Symptomatic knee osteoarthritis (KOA)

Symptomatic KOA was defined as both doctor-diagnosed arthritis and self-reported pain in the knee joint. The presence of knee joint pain was assessed based on the following question: “Are you often troubled by pain in any part of your body?” and “In what part of your body do you feel pain?” [28].

#### Education

Education is categorized as “no formal education”, “Did not finish primary school but capable of reading or writing”, “Home School”, “elementary school”, “middle school”, “high school”, “Vocational school”, “Two/Three Year College / Associate degree”, “Four Year College / Bachelor’s degree”, “Post-graduate, Master’s degree”, “Post-graduate, Doctoral degree/Ph.D.”

#### Depression

Ten depression questions of the Center for Epidemiologic Studies-Depression Scale (CES-D) were used in CHARLS, including two positive questions and eight negative questions. Each question was based on a four-point Likert scale: rarely, some days, occasionally and most of the time. The depression score is the sum of scores for these questions, varying from 0 to 30 points. The depression variable is defined as “yes” if the CES-D score is higher than 12 and the other is defined as “no” [29].

#### Physical activity (PA)

The questions assessing physical activity of CHARLS were similar to the short version of International Physical Activity Questionnaire (IPAQ), which is highly recognized questionnaire to measure PA [20]. Although the IPAQ was originally designed for people up to 69 years old, it was believed to be a useful tool for assessing PA among elderly adults [30]. But there were three differences between IPAQ and CHARLS questionnaire. First, the information of PA was collected during “a usual week” instead of “the last 7 days”. Second, no information was collected about sedentariness. Third, CHARLS reported discrete time instead of continuous time [31]. The duration of physical activity is categorized into 4 groups including < 30 min, 30 min – 2 h, 2 – 4 h, ≥ 4 h.

We used the median of each group as the time duration of each intensity level, while the group “more than 4 hours” were identified as 4 h. The amount of different intensity levels were then calculated using the metabolic equivalent (MET) as IPAQ [32].

#### Body mass index (BMI)

Weight (kg) and height (cm) were measured without shoes. BMI is calculated as the individual’s weight divided by the square of the height (kg/m^2^), and BMI group is categorized into 6 groups (< 18.5 kg/m^2^, 18.5–22.9 kg/m^2^, 23.0–27.49 kg/m^2^, ≥ 27.5 kg/m^2^) [33].

#### Hearth problems and memory related disease

Heart problems include heart attack, coronary heart disease, angina, and congestive heart failure. Memory related disease include Alzheimer’s disease, brain atrophy, and Parkinson’s disease.

#### Age

Age is categorized into 4 groups (age < = 49 years old, 50 years old < = age < = 59 years old, 60 years old < = age < = 69 years old, age > = 70 years old).

### Statistical analysis

The data are expressed as frequencies (percentage) and means ± standard deviations (SDs) for baseline characteristics. T-test was performed for continuous variables, chi-square test or Fisher’s exact test was performed for unordered categorical variables and Wilcoxon rank sum test was performed for ordered categorical variables to compare the difference in the baseline characteristics.

First, a stepwise logistic regression including forward and backward selections was used. To adjust for confounding factors, variables with a univariable *P*-value < 0.05 were included in the multivariable logistic regression model. Second, we used the least absolute shrinkage and selection operator (LASSO) [34]. We used a 10-fold cross validation to select an optimal hyperparameter (lambda) [34]. In cross validation, optimal lambda was chosen as the most regularized model [34].

Two-sided *P* values < 0.05 were considered statistically significant. All analyses were performed using STATA version 15.0 (STATA Corporation, College Station, TX).

## Results

### Univariable analysis of risk factors of fall

After completing univariable analysis between no-fall group and fall group, participants with older age (*P* < 0.001), obese (*P* = 0.007), female (*P* < 0.001), weak grip strength (*P* < 0.001), lower education level (*P* < 0.001) had a higher risk of fall. The participants who had chronic diseases including systematic knee osteoarthritis (*P* < 0.001), depression (*P* < 0.001), chronic lung disease (*P* < 0.001), hypertension (*P* = 0.001), heart problems (*P* = 0.001), ischemic stroke (*P* < 0.001), kidney disease (*P* < 0.001), gastrointestinal disease (*P* < 0.001) and rheumatoid arthritis (*P* < 0.001) were at a higher risk of fall. The results are shown in Table 1.

**Table 1:** Univariable analysis of risk factors of fall

Variables	Categories	No-fall group	Fall group	*P* value
Gender	male	3406 (49.5%)	980 (40.9%)	<0.001
	female	3475 (50.5%)	1416 (59.1%)	
Symptomatic knee osteoarthritis	yes	500 (7.3%)	301 (12.6%)	<0.001
	none	6386 (92.7%)	2097 (87.4%)	
Depression	yes	1455 (22.2%)	774 (34.1%)	<0.001
	none	5112 (77.8%)	1493 (65.9%)	
Grip strength	normal	6163 (89.5%)	1997 (83.3%)	<0.001
	weak	723 (10.5%)	401 (16.7%)	
Education	no formal education	1750 (25.4%)	780 (32.5%)	<0.001
	did not finish primary school	1215 (17.6%)	445 (18.6%)	
	sishu	24 (0.3%)	11 (1.6%)	
	elementary school	1610 (23.4%)	522 (21.8%)	
	middle school	1501 (21.8%)	435 (18.1%)	
	high school	548 (8.0%)	124 (5.2%)	
	vocational school	130 (1.9%)	62 (2.6%)	
	two/three-year college	80 (1.2%)	14 (0.6%)	
	four-year college	25 (0.3%)	4 (0.2%)	
	post graduate	1 (0.01%)	1 (0.04%)	
Hypertension	yes	1490 (21.7%)	597 (25.1%)	0.001
	none	5363 (78.3%)	1786 (74.9%)	
Chronic lung diseases	Yes	597 (8.7%)	276 (11.6%)	<0.001
	none	6263 (91.3%)	2109 (88.4%)	
Heart problems	Yes	697 (10.2%)	299 (12.5%)	0.001
	none	6152 (89.8%)	2086 (87.5%)	
Ischemic stroke	Yes	91 (1.3%)	68 (2.8%)	<0.001
	none	6777 (98.7%)	2322 (97.2%)	
Kidney disease	Yes	371 (5.4%)	191 (8.0%)	<0.001
	none	6469 (94.6%)	2189 (92.0%)	
Gastrointestinal disease	Yes	1457 (21.2%)	591 (24.7%)	<0.001
	none	5408 (78.8%)	1799 (75.3%)	
Rheumatoid arthritis	Yes	2069 (30.1%)	976 (40.8%)	<0.001
	none	4806 (69.9%)	1417 (59.2%)	
BMI (kg/m^2^)	< 18.5	401 (5.9%)	167 (7.1%)	0.007
	18.5 – 22.9	2811 (41.2%)	1014 (42.8%)	
	23.0 – 27.49	2603 (38.2%)	870 (36.7%)	
	>=27.5	1005 (14.7%)	317 (13.4%)	
Age (years old)	<= 49	1626 (23.6%)	413 (17.3%)	<0.001
	50 - 59	2520 (36.6%)	790 (33.0%)	
	60 - 69	1915 (27.8%)	750 (31.3%)	
	>= 70	816 (11.9%)	440 (18.4%)	

*BMI* Body mass index

### Univariable analysis of risk factors of weak grip strength

As reported in Table 2, the distribution of gender, BMI, age, education, living area, proportion of patients with hypertension, chronic lung diseases, heart problems, ischemic stroke, memory related disease, rheumatoid arthritis, asthma, depression, levels of physical activity, frequencies of drinking alcohol, and history of symptomatic knee osteoarthritis showed significant differences (*P* < 0.05) between normal grip strength group and weak grip strength group.

**Table 2:** Univariable analysis of risk factors of weak grip strength

	Normal grip strength	Weak grip strength	*P* value
Gender			
Female	3750 (53.0%)	776 (57.1%)	0.007
Male	3319 (47.0%)	584 (42.9%)	
Education			
No formal education	1681 (23.8%)	506 (37.2%)	<0.001
Did not finish primary school	1275 (18.0%)	292 (21.5%)	
Sishu	25 (0.4%)	7 (0.5%)	
Elementary school	1646 (23.3%)	325 (23.9%)	
Middle school	1669 (23.6%)	168 (12.3%)	
High school	558 (7.9%)	36 (2.6%)	
Vocational school	122 (1.7%)	23 (1.7%)	
Two/three-year college	77 (1.1%)	3 (2.2%)	
Four-year college	22 (0.3%)	1 (0.07%)	
Post graduate	1 (0.01%)	0 (0.0%)	
Living area			
Rural	3494 (83.2%)	783 （89.3%）	<0.001
Urban	708 (16.8%)	94 (10.7%)	
Hypertension			
Yes	1519 (21.6%)	390 (28.9%)	<0.001
None	5517 (78.4%)	959 (71.1%)	
Chronic lung diseases			
Yes	623 (8.8%)	178 (13.1%)	<0.001
None	6417 (91.2%)	1179 (86.9%)	
Heart problems			
Yes	701 (10.0%)	186 (13.8%)	<0.001
None	6330 (90.0%)	1164 (86.2%)	
Ischemic stroke			
Yes	104 (1.5%)	39 (2.9%)	<0.001
None	6945 (98.5%)	1321 (97.1%)	
Memory related disease			
Yes	68 (1.0%)	23 (1.7%)	0.018
None	6977 (90.0%)	1337 (98.3%)	
Rheumatoid arthritis			
Yes	2287 (32.4%)	524 (38.5%)	<0.001
None	4766 (67.6%)	837 (61.5%)	
Asthma			
Yes	216 (3.1%)	71 (5.2%)	<0.001
None	6822 (96.9%)	1287 (94.8%)	
Physical activity			
Low level	248 (8.9%)	40 (7.8%)	0.049
Moderate level	438 (15.8%)	103 (20.0%)	
High level	2093 (75.3%)	371 (72.2%)	
Drinking alcohol			
Always	1844 (26.1%)	308 (22.6%)	<0.001
Seldom	578 (8.2%)	82 (6.0%)	
None	4650 (65.8%)	971 (71.3%)	
BMI (kg/m^2^)			
< 18.5	285 (4.1%)	143 (10.7%)	<0.001
18.5–22.9	2766 (39.4%)	615 (22.9%)	
23.0–27.49	2872 (40.9%)	429 (16.0%)	
>=27.5	1097 (15.6%)	153 (5.7%)	
Symptomatic knee osteoarthritis			
Yes	580 (8.2%)	171 (12.6%)	<0.001
None	6497 (91.8%)	1190 (87.4%)	
Depression			
Yes	2221 (32.9%)	592 (46.6%)	<0.001
None	4538 (67.1%)	678 (53.4%)	
Age (years old)			
<=49	1792 (25.4%)	117 (8.6%)	<0.001
50-59	2842 (40.2%)	346 (25.5%)	
60-69	1941 (27.5%)	522 (38.4%)	
>=70	492 (7.0%)	373 (27.5%)	

*BMI* Body mass index

### Stepwise logistic regression and LASSO regression of fall

**Fig. 1:**
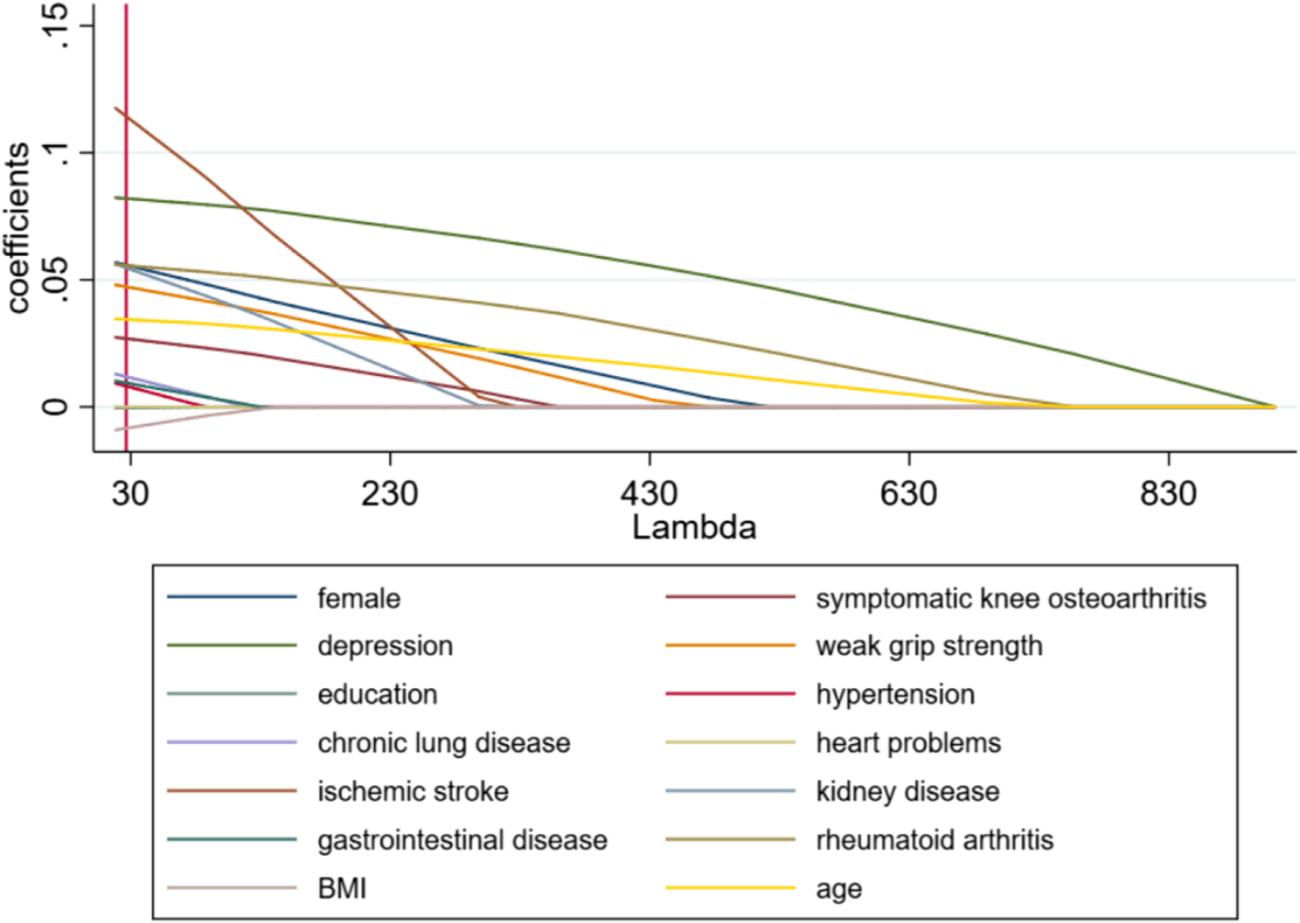
LASSO regression model of fall: Shrinkage of coefficients by hyperparameter (λ)

According to the univariable analysis, logistic regression was done. Firstly, we compared the variables between no-fall group and fall group using logistic regression (Supplementary Table 1**)**. After further adjusted for gender, age, grip strength, and chronic disease, female (aOR = 1.382, 95% CI = 1.240–1.541; *P* < 0.001), depression (aOR = 1.516, 95% CI = 1.354–1.699; *P* < 0.001), weak grip strength (aOR = 1.264, 95% CI = 1.086–1.471; *P* = 0.003), ischemic stroke (aOR = 1.786, 95% CI = 1.259–2.534; *P* = 0.001), kidney disease (aOR = 1.344, 95% CI = 1.102–1.640; *P* = 0.004), rheumatoid arthritis (aOR = 1.354, 95% CI = 1.206–1.520; *P* < 0.001), older age (aOR = 1.212, 95% CI = 1.144–1.283; *P* < 0.001) were considered as significant risk factors based on the OR values (OR > 1). Then, stepwise logistic regression was used to cut down variables. The forward and backward selections showed the same results. The results are shown in Table 3. The risk factors included female (aOR = 1.376, 95% CI = 1.244–1.523; *P* < 0.001), ischemic stroke (aOR = 1.786, 95% CI = 1.263–2.524; *P* = 0.001), depression (aOR = 1.559, 95% CI = 1.396–1.742; *P* < 0.001), weak grip strength (aOR = 1.285, 95% CI = 1.105–1.494; *P* = 0.001), older age (aOR = 1.227, 95% CI = 1.163–1.294; *P* < 0.001), rheumatoid arthritis (aOR = 1.410, 95% CI = 1.270–1.566; *P* < 0.001), kidney disease (aOR = 1.383, 95% CI = 1.136–1.682; *P* = 0.001). The results of the LASSO regression model are summarized in Fig. 1. In cross validation, the optimal λ was 26.572. At this level, the coefficient estimates of all the risk factors were not shrunken by LASSO towards zero. Logistic regression, stepwise regression and LASSO regression were separately preformed in male group and female group. The results were shown in Supplementary Tables 2, 3, 4, 5 and Supplementary Figs. 1, 2.

**Table 3:** Stepwise logistic regression results of risk factors of fall

Fall	OR	*P* value	95% CI
Depression	1.559	< 0.001	1.396–1.742
Age^a^	1.227	< 0.001	1.163–1.294
Rheumatoid arthritis	1.410	< 0.001	1.270–1.566
Female	1.376	< 0.001	1.244–1.523
Weak grip strength	1.285	0.001	1.105–1.494
Ischemic stroke	1.786	0.001	1.263–2.524
Kidney disease	1.383	0.001	1.136–1.682

*OR* Odds ratio, *CI* Confidence Interval, ^a^age was included in this model as ordered categorical variables

### Stepwise logistic regression and LASSO regression of weak grip strength

**Fig. 2:**
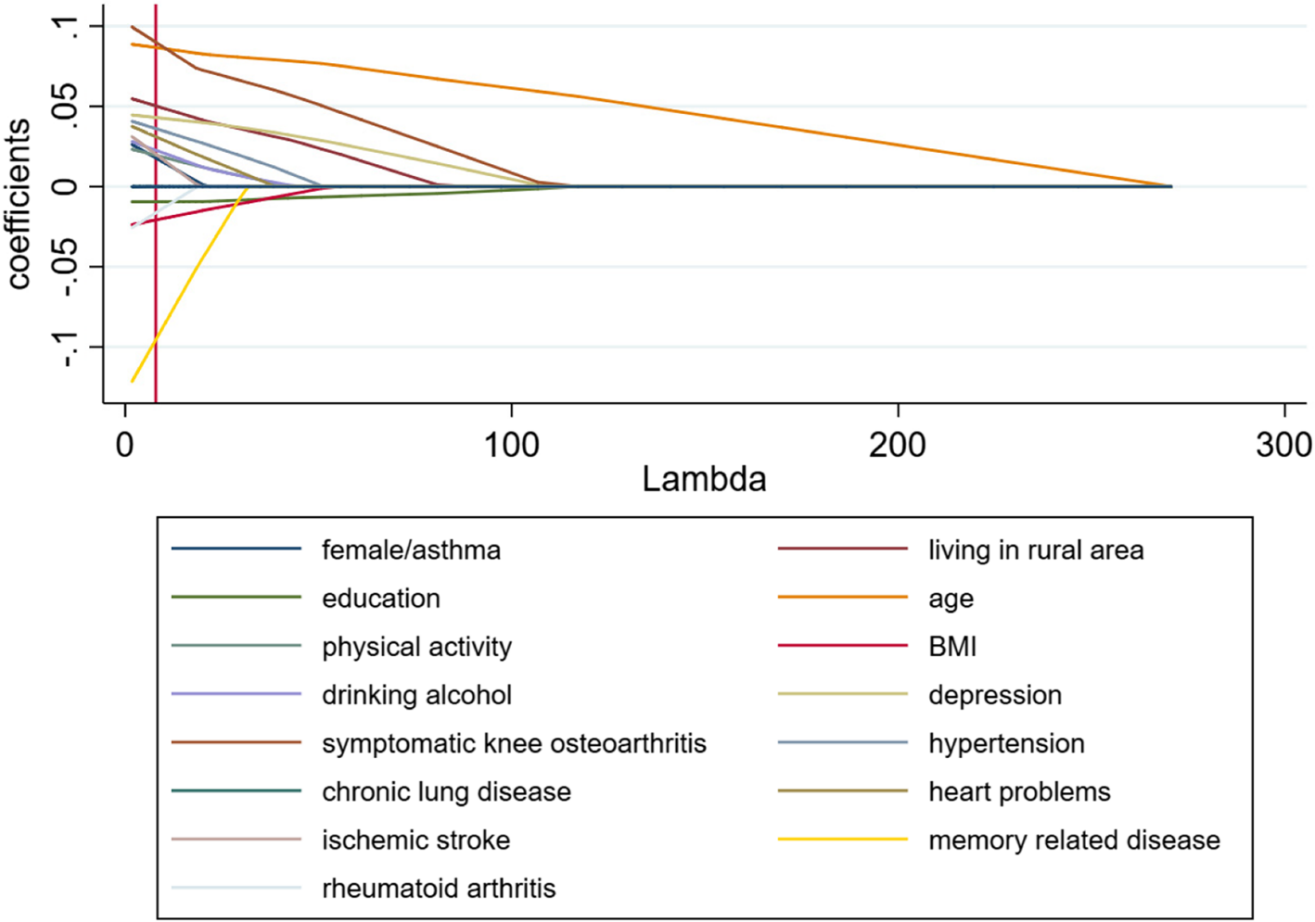
LASSO regression model of weak grip strength: Shrinkage of coefficients by hyperparameter (λ)

Firstly, we compared the variables between weak grip strength and normal grip strength group, as shown in Supplementary Table 6. For the weak grip-strength population, risk factors included living in rural area (aOR = 1.703, 95% CI = 1.033–2.806; *P* = 0.037), older age (aOR = 1.993, 95% CI = 1.670–2.378; *P* < 0.001), symptomatic knee osteoarthritis (aOR = 1.912, 95% CI = 1.156–3.164; *P* = 0.012) based on the OR values (OR > 1). Then the stepwise logistic regression results showed as Table 4. Symptomatic knee osteoarthritis (aOR = 1.755, 95% CI = 1.158–2.661; *P* = 0.008), living in rural area (aOR = 2.056, 95% CI = 1.290–3.277; *P* = 0.002), depression (aOR = 1.523, 95% CI = 1.116–2.078; *P* = 0.008), being older (aOR = 2.116, 95% CI = 1.801–2.486; *P* < 0.001) showed significant influence on grip strength. The results of the LASSO regression model are summarized in Fig. 2. In cross validation, the optimal λ was 7.889. At this level, the coefficient estimates of all the risk factors were not shrunken by LASSO towards zero. Logistic regression, stepwise regression and LASSO regression of weak grip strength were separately preformed in male group and female group. The results were shown in Supplementary Tables 7, 8, 9, 10 and Supplementary Figs. 3, 4.

**Table 4:** Stepwise logistic regression results of weak grip strength

Grip strength	OR	P value	95% CI
Symptomatic knee osteoarthritis	1.755	0.008	1.158–2.661
Living in rural area	2.056	0.002	1.290–3.277
Depression	1.523	0.008	1.116–2.078
Age^b^	2.116	< 0.001	1.801–2.486

^a^*OR* Odds ratio, *CI* Confidence Interval; ^b^age was included in this model as ordered categorical variables

## Discussion

The primary purpose of this study was to determine the risk factors of fall and weak grip strength. We used data from CHARLS and observed that fall and weak grip strength had many similar risk factors. Depression, age, symptomatic knee osteoarthritis were risk factors of both weak grip strength and fall. Furthermore, female with ischemic stroke and kidney disease had higher risk of fall. People living in rural area had higher risk of weak grip strength.

Depression was always accompanied by several psychophysiological changes, such as disturbances in appetite, sleep [35, 36]. According to WHO, 350 million people suffered from depression all over the world [36]. Depression can also not be considered a normal procedure of ageing. Many adolescents exhibited depressive symptoms. Some researchers found no relationship between weak grip strength and depression [37–39], while other researchers suggested a relationship between depressive symptoms with weak grip strength [40–43]. Our study found that depression is a risk factor of weak grip strength. Depressed individuals may perform limited physical activities, causing weaker muscle strength. Especially, depressed participants may not fully squeeze the handle due to lack of motivation [44]. Therefore, depressed participants were recorded with weak grip strength results.

Considering fall, as we showed, depressed people got higher risks of fall. The actual mechanism why depressive mood predisposed to falls is unknown. Maybe depressed people can’t take care of themselves and thus perform high risk behavior to fall more often [45]. In particular, depressed people may show impaired protective responses, leading to increased risk of injury [45].

Our study showed no relationship between body mass with fall as the result of logistic regression. But many studies [46, 47] had shown that disabled older people, especially female with lower BMI, are more likely to fall. A low body mass may indicate poor nutritional status or health status [47, 48]. There is also some evidence reflecting that in female, a high body mass may increase the concentration of estrogen, which may have a positive effect on bone construction [49, 50]. Our research did not show this. It may be that our study mixed normal and disabled people. A low body mass may reflect poor nutritional status or may only indicate slimness. Moreover, the body mass of Chinese people is commonly lower than western people. We got more normal BMI or just overweight people.

PA contributes to muscle strength a lot and the mechanisms include reduced apoptosis, reduced oxidative stress, anti-inflammation, improved insulin-glucose dynamics, enhanced quality and quantity of muscle proteins and mitochondria, skeletal muscle hypertrophy, positive neuromuscular adaptations, and enhanced muscle blood supply [51].

Some evidences revealed that high PA indicated higher muscle mass, muscle strength and better physical performance compared with low and moderate PA levels [52, 53]. But others did not suggest such association [54, 55]. In a systematic review, Beckwée et al. concluded that exercise contributed to improving muscle strength [56]. Conversely, Yoshimura et al. suggested that exercise had no significant effects on muscle strength [57]. It is still controversial. Also, some studies showed that physical activity associates positively with lower-limb strength but less with grip strength [58, 59]. Resistance exercise is currently the primary recommendation for enhancing muscle strength [60, 61].WHO recommends that at least 150 min of moderate aerobic PA or 75 min of vigorous aerobic PA per week for older adults (65 years and above) [62].

Our study showed no association between grip strength with physical activities. On one hand, our definition of PA is based on IPAQ, a reflect of daily activities. On the other hand, there are no sedentary time related questions in CHARLS questionnaire. Thus, targeted exercise is not involved, which is considered to improve muscle strength.

PA restriction could be beneficial for safety by reducing risk exposure [63]. However, long-term PA restriction on various activities leads to increased risk of falls [63]. Higher levels of physical activity were associated with higher frequencies of fall [64].

Muscle strength of lower limbs is considered as an important factor affecting individual fall [65]. In our study, we found that grip strength can also reflect the risk of fall in Chinese people. In clinical practice, grip strength is easier to measure [65]. Doctors can evaluate the muscle strength via grip strength to predict the risk of fall.

In addition, we found that older age, female, ischemic stroke, rheumatoid arthritis, kidney disease were risk factors of fall. Ischemic stroke and rheumatoid arthritis can directly affect balance and gait [66, 67]. And kidney disease affects the homeostasis [68]. Thus, these people get worse muscle function.

The strength of this study is that our study was based on CHARLS, involving 150 countries/districts, 450 villages/urban communities across the country and using proper sampling strategy [20]. Thus, the sample could be well representative. However, some limitations still exist. First, CHARLS was a short follow-up survey. This still requires long-term follow-up to explore risk factors for fall and weak grip strength. Second, many covariates such as chronic diseases in CHARLS were self-reported, which may increase the risk of residual confounding. Third, because data involves health information and personal information, people may withhold relevant information. Some information is to make people recall some life and health conditions more than one year ago. These all could lead to bias. Finally, our research was based on the previous studies. The variables involved in this study were based on the previous studies and our clinical experience. There may be some influencing factors that were not included in our study that led to bias.

### Conclusion

Age and depression were risk factors of both weak grip strength and fall. Other risk factors, including female, ischemic stroke, rheumatoid arthritis, and kidney disease were risk factors of fall; living in rural area and symptomatic knee osteoarthritis were risk factors of weak grip strength. Through our research involving thousands of participants, we found that people who fell and people with weak grip strength have many similar characteristics. It is of great value for us to unify the management of such people with common characteristics in the future and reduce the cost of health service systems. At the same time, personalized management of people with weak grip strength or people who fell should not be lost.

## Supplementary Information

**Additional file 1: Table S3.** CASP checklist – Risk of Bias for Cohort and Case control studies. Summarized responses for risk of bias of cohort and case control studies.

## Data Availability

Details of the CHARLS data are available from http://charls.pku.edu.cn/pages/data/111/zh-cn.html.

## Abbreviations

CHARLS: China Health and Retirement Longitudinal Study
PPS: Probability proportionate to size
KOA: Knee osteoarthritis
PA: Physical activity
IPAQ: International Physical Activity Questionnaire
MET: Metabolic equivalent
BMI: Body mass index
SDs: Standard deviations
OR: Odds ratio
S.E.: Standard error
CI: Confidence Interval
LASSO: Least absolute shrinkage and selection operator
