# Supplementary material for: Influencing factors of weak grip strength and fall: a study based on the China Health and Retirement Longitudinal Study (CHARLS)
Source: BMC Public Health. 2022 Dec 13;22:2337. doi: 10.1186/s12889-022-14753-x (PMC9749312; doi:10.1186/s12889-022-14753-x)
Supplement: Supplementary file 1 — Additional file 1: Table S3. CASP checklist – Risk of Bias for Cohort and Case control studies. Summarized responses for risk of bias of cohort and case control studies. [file 12889_2022_14753_MOESM1_ESM.pdf]

Supplementary Table 1. Logistic regression results of risk factors of fall

fall	OR	*P* value	95% CI
female	1.382	<0.001	1.240-1.541
symptomatic knee osteoarthritis	1.113	0.258	0.924-1.342
depression	1.516	<0.001	1.354-1.699
weak grip strength	1.264	0.003	1.086-1.471
education**	0.996	0.779	0.967-1.025
hypertension	1.067	0.304	0.943-1.209
chronic lung disease	1.081	0.361	0.915-1.278
heart problems	0.979	0.801	0.833-1.152
ischemic stroke	1.786	0.001	1.259-2.534
kidney disease	1.344	0.004	1.102-1.640
gastrointestinal disease	1.066	0.301	0.944-1.203
rheumatoid arthritis	1.354	<0.001	1.206-1.520
BMI**	0.945	0.091	0.886-1.009
age**	1.212	<0.001	1.144-1.283

BMI-body mass index; OR-odds ratio; CI-Confidence Interval; **education, BMI, age were included in this model as ordered categorical variables

Supplementary Table 2. Logistic regression results of fall in male group

fall	OR	*P* value	95% CI
symptomatic knee osteoarthritis	1.579	0.003	1.168 - 2.133
depression	1.589	0.000	1.326 - 1.904
weak grip strength	1.741	0.000	1.389 - 2.183
education**	0.981	0.406	0.937 - 1.027
hypertension	1.037	0.717	0.853 - 1.261
chronic lung disease	1.117	0.352	0.885 - 1.412
heart problems	1.020	0.883	0.784 - 1.328
ischemic stroke	1.851	0.013	1.137 - 3.013
kidney disease	1.123	0.427	0.844 - 1.493
gastrointestinal disease	1.048	0.621	0.869 - 1.264
rheumatoid arthritis	1.313	0.003	1.098 - 1.570
BMI**	0.886	0.025	0.797 - 0.985
age**	1.079	0.092	0.987 - 1.180

BMI-body mass index; OR-odds ratio; CI-Confidence Interval; **education, age, BMI were included in this model as ordered categorical variables

Supplementary Table 3. Stepwise logistic regression results of fall in male group

fall	OR	*P* value	95% CI
symptomatic knee osteoarthritis	1.630	0.001	1.210 - 2.196
depression	1.627	0.000	1.361 - 1.944
weak grip strength	1.762	0.000	1.407 - 2.206
rheumatoid arthritis	1.339	0.001	1.122 - 1.598
BMI**	0.883	0.016	0.798 - 0.977
ischemic stroke	1.868	0.011	1.156 - 3.019
age**	1.097	0.031	1.008 - 1.193

BMI-body mass index; OR-odds ratio; CI-Confidence Interval; **age, BMI were included in this model as ordered categorical variables

Supplementary Figure 1. LASSO regression model of fall in male group: Shrinkage of coefficients by hyperparameter (λ)

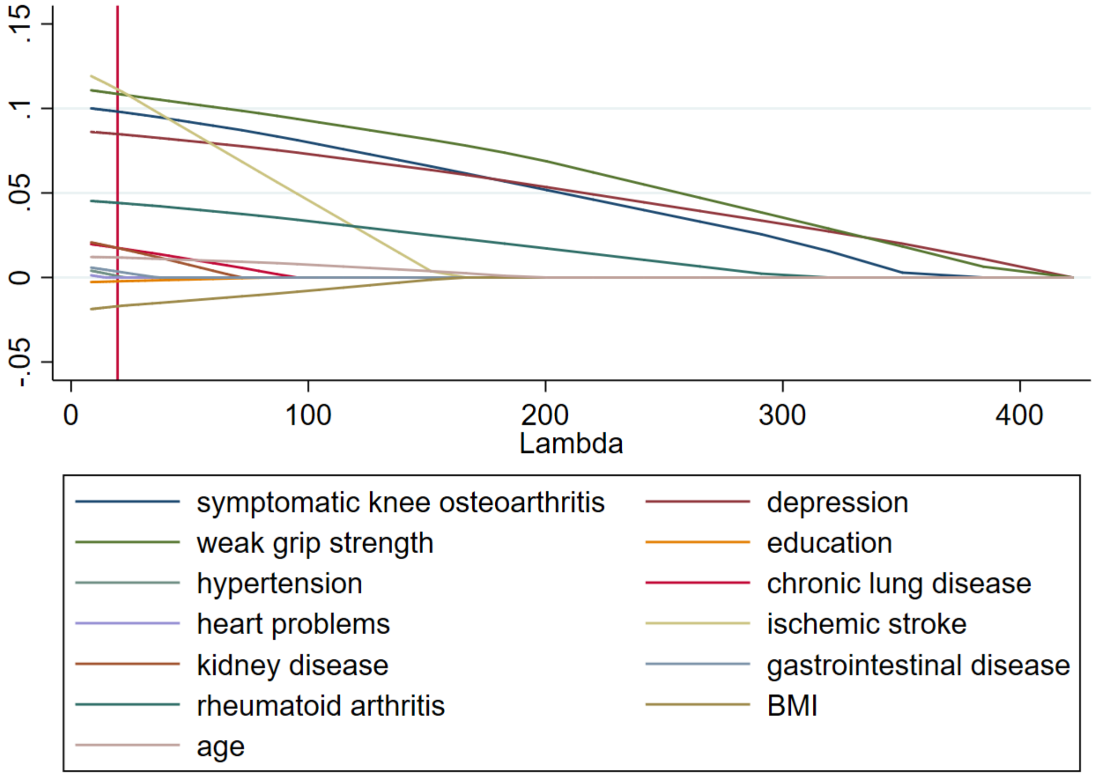

*The optimal lambda was 19.591

Supplementary Table 4. Logistic regression results of fall in female group

fall	OR	*P* value	95% CI
symptomatic knee osteoarthritis	0.916	0.468	0.722 - 1.162
depression	1.476	0.000	1.274 - 1.709
weak grip strength	0.988	0.906	0.804 - 1.213
education**	1.008	0.676	0.970 - 1.048
hypertension	1.081	0.349	0.919 - 1.272
chronic lung disease	1.040	0.752	0.817 - 1.324
heart problems	0.969	0.763	0.787 - 1.192
ischemic stroke	1.758	0.029	1.058 - 2.922
kidney disease	1.598	0.001	1.204 - 2.121
gastrointestinal disease	1.095	0.268	0.933 - 1.285
rheumatoid arthritis	1.358	0.000	1.165 - 1.583
BMI**	0.988	0.773	0.909 - 1.074
age**	1.318	0.000	1.223 - 1.420

BMI-body mass index; OR-odds ratio; CI-Confidence Interval; **education, age, BMI were included in this model as ordered categorical variables

Supplementary Table 5. Stepwise logistic regression results of fall in female group

fall	OR	*P* value	95% CI
ischemic stroke	1.770	0.027	1.067 - 2.935
depression	1.473	0.000	1.278 - 1.698
age	1.316	0.000	1.229 - 1.408
kidney disease	1.615	0.001	1.220 - 2.137
rheumatoid arthritis	1.347	0.000	1.173 - 1.547

OR-odds ratio; CI-Confidence Interval; ** age were included in this model as ordered categorical variables

Supplementary Figure 2. LASSO regression model of fall in female group: Shrinkage of coefficients by hyperparameter (λ)

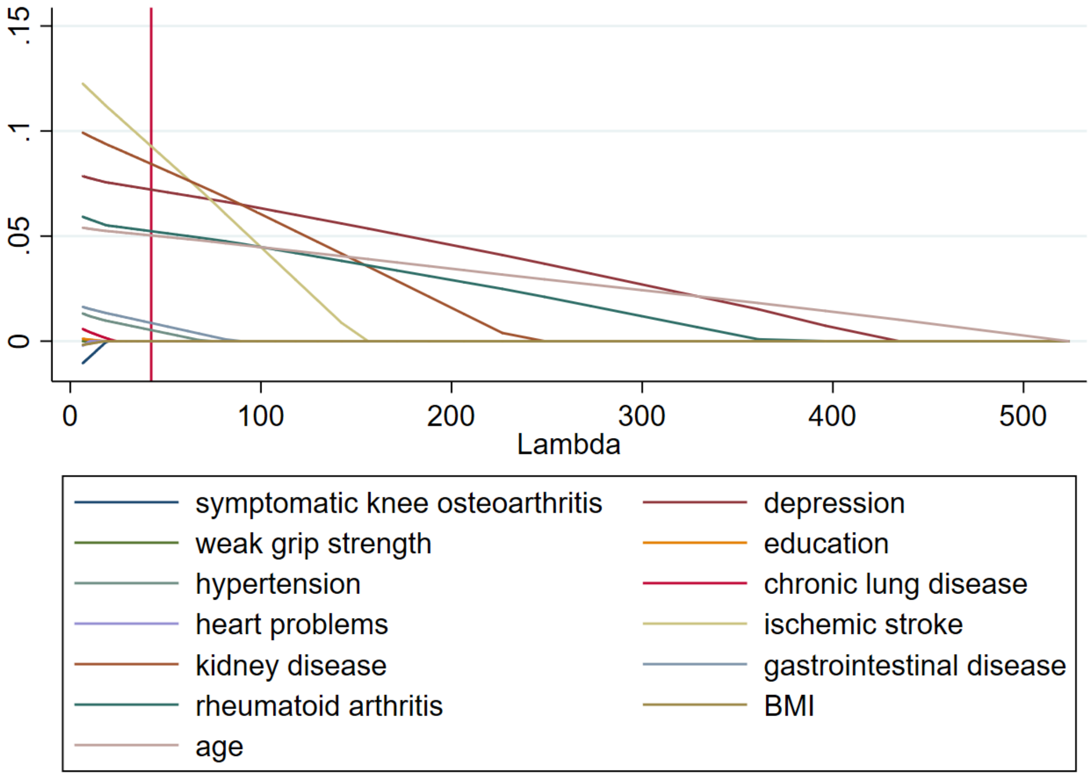

*The optimal lambda was 42.466

Supplementary Table 6. Logistic regression results of weak grip strength

weak grip strength	OR	*P* value	95% CI
female	1.187	0.349	0.829-1.701
living in rural area	1.703	0.037	1.033-2.806
education**	0.915	0.063	0.833-1.005
age**	1.993	<0.001	1.670-2.378
physical activity level**	1.261	0.063	0.988-1.611
BMI**	0.834	0.071	0.686-1.015
drinking alcohol	1.217	0.107	0.958-1.545
depression	1.366	0.059	0.988-1.887
symptomatic knee osteoarthritis	1.912	0.012	1.156-3.164
hypertension	1.370	0.081	0.962-1.953
chronic lung disease	1.031	0.904	0.629-1.689
heart problems	1.335	0.196	0.862-2.068
ischemic stroke	1.268	0.569	0.561-2.866
memory related problems	0.390	0.377	0.049-3.142
rheumatoid arthritis	0.791	0.214	0.547-1.144
asthma	0.985	0.968	0.465-2.085

BMI-body mass index; OR-odds ratio; CI-Confidence Interval; **education, age, BMI, physical activity level were included in this model as ordered categorical variables

Supplementary Table 7. Logistic regression results of weak grip strength in male group

weak grip strength	OR	*P* value	95% CI
living in rural area	1.970	0.119	0.840 - 4.625
education**	0.951	0.512	0.817 - 1.106
age**	2.474	0.000	1.806 - 3.387
physical activity level**	1.257	0.302	0.814 - 1.940
BMI**	0.731	0.074	0.519 - 1.031
drinking alcohol	1.267	0.121	0.939 - 1.710
depression	1.058	0.851	0.589 - 1.899
symptomatic knee osteoarthritis	2.689	0.038	1.055 - 6.856
hypertension	2.073	0.018	1.134 - 3.790
chronic lung disease	0.749	0.467	0.343 - 1.635
heart problems	1.482	0.302	0.702 - 3.125
ischemic stroke	0.951	0.942	0.246 - 3.673
memory related problems	0.618	0.663	0.071 - 5.371
rheumatoid arthritis	0.780	0.436	0.417 - 1.458
asthma	1.296	0.653	0.418 - 4.022

BMI-body mass index; OR-odds ratio; CI-Confidence Interval; **education, BMI, age, physical activity level were included in this model as ordered categorical variables

Supplementary Table 8. Stepwise logistic regression results of weak grip strength in male group

weak grip strength	OR	*P* value	95% CI
living in rural area	2.601	0.020	1.161 - 5.830
symptomatic knee osteoarthritis	2.361	0.027	1.104 - 5.045
age**	2.571	0.000	1.920 - 3.442
hypertension	1.898	0.016	1.125 - 3.204

**age was included in this model as ordered categorical variables

Supplementary Figure 3. LASSO regression model of weak grip strength in male group: Shrinkage of coefficients by hyperparameter (λ)

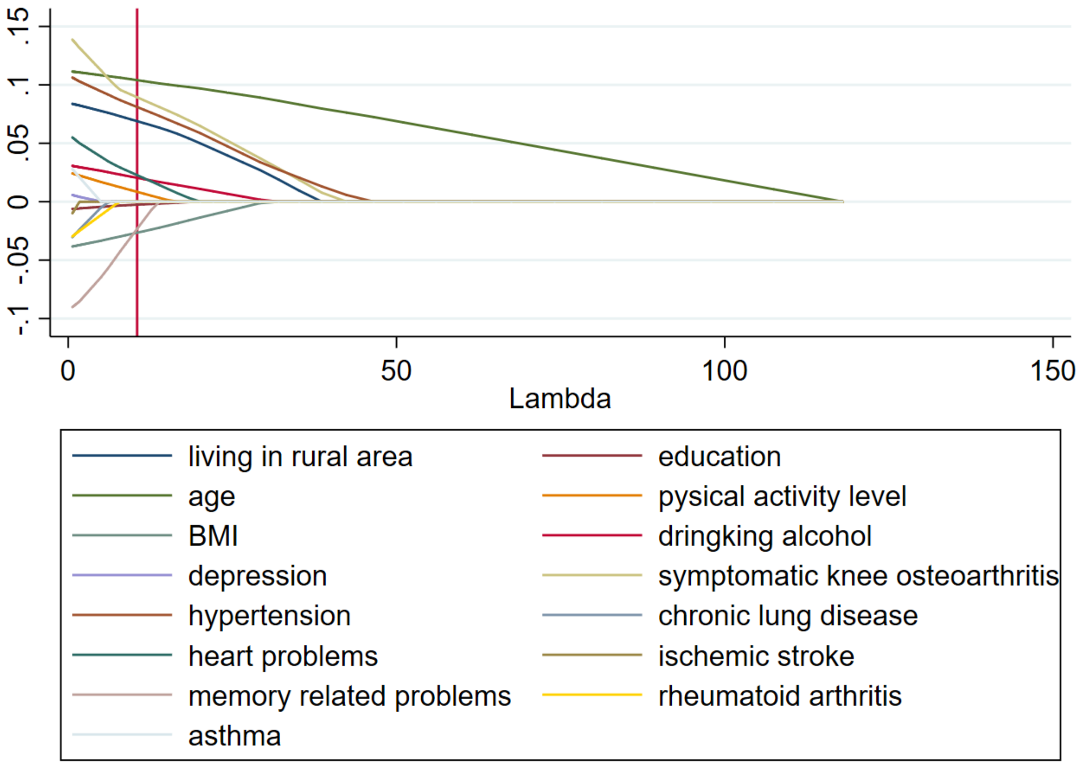

*The optimal lambda was 10.5

Supplementary Table 9. Logistic regression results of weak grip strength in female group

weak grip strength	OR	*P* value	95% CI
living in rural area	1.498	0.201	0.807 - 2.782
education**	0.879	0.041	0.777 - 0.995
age**	1.804	0.000	1.448 - 2.247
physical activity level**	1.251	0.142	0.928 - 1.687
BMI**	0.887	0.329	0.696 - 1.129
drinking alcohol	1.128	0.598	0.721 - 1.766
depression	1.560	0.027	1.052 - 2.315
symptomatic knee osteoarthritis	1.710	0.086	0.926 - 3.159
hypertension	1.081	0.731	0.692 - 1.689
chronic lung disease	1.247	0.509	0.648 - 2.400
heart problems	1.251	0.428	0.719 - 2.179
ischemic stroke	1.568	0.402	0.548 - 4.486
rheumatoid arthritis	0.817	0.391	0.514 - 1.298
asthma	0.809	0.693	0.282 - 2.322

BMI-body mass index; OR-odds ratio; CI-Confidence Interval; **education, BMI, age, physical activity level were included in this model as ordered categorical variables

Supplementary Table 10. Stepwise logistic regression results of weak grip strength in female group

weak grip strength	OR	*P* value	95% CI
depression	1.801	0.002	1.241 - 2.613
education**	0.855	0.008	0.762 - 0.960
age**	1.793	0.000	1.463 - 2.197

**education, age were included in this model as ordered categorical variables

Supplementary Figure 4. LASSO regression model of weak grip strength in female group: Shrinkage of coefficients by hyperparameter (λ)

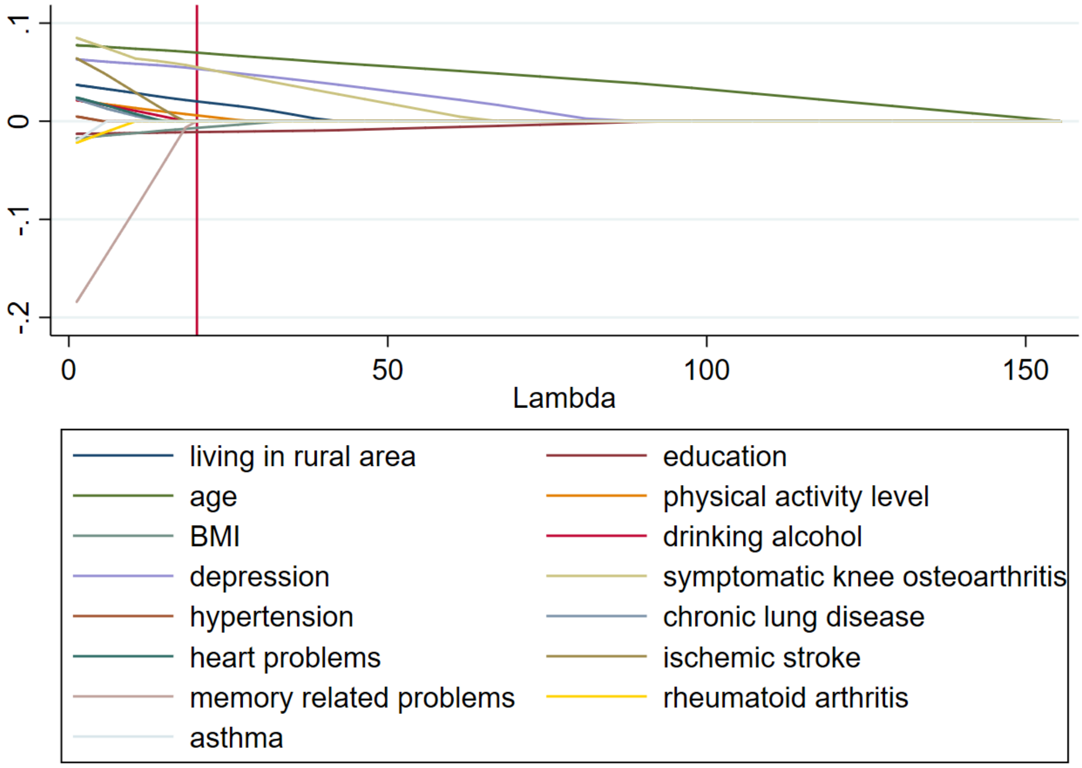

*The optimal lambda was 20.08
